# Effect of mango seeds as an untraditional source of energy on the productive performance of dairy Damascus goats

**DOI:** 10.3389/fvets.2023.1058915

**Published:** 2023-02-14

**Authors:** Heba A. El-Sanafawy, Aristide Maggiolino, Ghada S. El-Esawy, Wasef A. Riad, Mohamed Zeineldin, Mohamed Abdelmegeid, Rabiha Seboussi, Lamiaa I. EL-Nawasany, Mona M. M. Y. Elghandour, Pasquale De Palo, Abdelfattah Z. M. Salem

**Affiliations:** ^1^Agricultural Research Center, Animal Production Research Institute, Giza, Egypt; ^2^Department of Veterinary Medicine, University of Bari A. Moro, Bari, Italy; ^3^Department of Animal Medicine, College of Veterinary Medicine, Benha University, Benha, Egypt; ^4^Department of Animal Medicine, College of Veterinary Medicine, Kafr-Elsheikh University, Kafr el-Sheikh, Egypt; ^5^Higher Colleges of Technology, Health Sciences Division, Abu Dhabi, United Arab Emirates; ^6^Facultad de Medicina Veterinaria y Zootecnia, Universidad Autónoma del Estado de México, Toluca, Mexico

**Keywords:** goats, digestibility, mango seeds, milk yield, yellow corn

## Abstract

Eighteen dairy Damascus goats weighing 38–45 kg live body weight and aged 3–4 years were divided into three groups according to their body weight, with six goats in each group. Yellow corn grain in their concentrate feed mixture was replaced with mango seeds (MS) at levels of 0% MS in group 1 (G1, control), 20% MS in group 2 (G2), and 40% MS in group 3 (G3). The digestibility coefficients of the organic matter, dry matter, crude fiber, crude protein, ether extract, nitrogen-free extract, and total digestible nutrients increased (*P* < 0.05) upon feeding MS to G2 and G3. The amounts of dry matter, total digestible nutrients, and digestible crude protein required per 1 kg 3.5% fat-corrected milk (FCM) were lower (*P* < 0.05) in G2 and G3 vs. G1. Actual milk and 3.5% FCM yield increased (*P* < 0.05) with the increasing MS dietary level. G2 and G3 had the highest significant (*P* < 0.05) total solids, total protein, non-protein nitrogen, casein, ash, fat, solids not fat, lactose, and calcium contents compared with G1. Replacing yellow corn grain with MS in G2 and G3 significantly (*P* < 0.05) decreased the cholesterol concentration and AST activity. Feeding MS increased the concentrations of caprioc, caprylic, capric, stearic, oleic, elaidic, and linoleic acids and decreased the concentrations of butyric, laueic, tridecanoic, myristic, myristoleic, pentadecanoic, heptadecanoic, cis-10-Heptadecanoic, cis-11-eicosenoic, linolenic, arachidonic, and lignoseric acids in the milk fat. The results show that the replacement of corn grain with MS improved the digestibility, milk yield, feed conversion, and economic efficiency, with no adverse effects on the performance of Damascus goats.

## Introduction

Mango residue, which accounts for 40–50% of the total fruit weight, has potential nutritional benefits in addition to its significant levels of essential minerals (1, 2). Recently, starch from fruit seeds, including mango, was found to possess good physicochemical properties in addition to a balanced amino acid concentration in the kernel (3). Mango seeds kernel (MSK), pulp, and peel contents are usually considered important bioactive materials because they contain antioxidant, anti-bacterial, and antiviral immune modulatory substances as well as lipids, and have a high protein content (4–6). MSK is considered a good source of carbohydrates (77%), fat (11%), proteins (6–7%), crude fiber (2%), and ash (2%) on a dry matter basis (7). MSK contains 210 mg/100 g magnesium, 170 mg/100 g calcium, and 368 mg/100 g potassium (8) as well as vitamin E (1.30 mg/100 g db), provitamin A (15.27 IU), vitamin C (0.56 mg/100 g db), and vitamin K (0.59 mg/100 g db) (9, 10).

Mango seeds kernel, as a nonconventional concentrate mixture, can greatly lower feed costs and can be safely integrated into lambs' rations without compromising the nutrient utilization, growth rate, feed intake, or blood profile (11, 12). The nutrient digestibility and consumption of sheep fed rice straw supplemented with mango kernel meal suggest that mango kernel meal can be used as an unusual feedstuff supplement during the lengthy dry season (13). Mango seeds kernel can also be used to replace up to half of the yellow corn in sheep rations without affecting feed intake, water metabolism, digestion coefficients, nitrogen balance, or ruminal fermentation (14). Replacement of corn grain with 30% MS in the diets of Damascus goat bucks showed positive effects on the productive performance and increased semen production (15). An economic evaluation of using whole mango meal in the diets of dairy goats, instead of corn, showed that the total feeding cost was reduced and the benefit-to-cost ratio improved (16). Furthermore, adding MS to mixed diets with alfalfa hay increased the diet's fermentation rate (17). Therefore, the objective of the present study was to investigate the effects of partial replacement of yellow corn grain with MS as a nontraditional source of energy on the productive performance of Damascus goats.

## Materials and methods

The present study was carried out at the Sakha Animal Production Research Station, which is part of the Animal Production Research Institute, Agricultural Research Center, Cairo, Egypt.

### Experimental animals

Eighteen dairy Damascus goats, weighing 38–45 kg live body weight and aged 3–4 years, were divided into three similar groups according to their body weight, with six goats in each group.

### Experimental diets

Three experimental diets were used to feed the goats during the experimental period (240 days). All diets contained a concentrate feed mixture [50% concentrate feed mixture (CFM)], fresh berseem (40%), and wheat straw (10%). Mango seeds were added instead of yellow corn grain in the CFM, at concentrations of 0% MS (G1, control), 20% (G2), and 40% (G3). The ingredients of the different CFMs are shown in Table 1.

**Table 1:** Formulation of different concentrate feed mixtures (CFM, % on DM basis).

**Ingredients**	**CFM1**	**CFM2**	**CFM3**
Yellow corn grains	30	24	18
Mango seeds	0	6	12
Wheat bran	40	40	40
Undecorticated cottonseed meal	24	24	24
Molasses	3	3	3
Limestone	2	2	2
Common salt	1	1	1

### Mango seeds preparation

Mango seeds consist of approximately 68% kernel, 29% shell, and 3% test (18). The mango seeds were soaked in water for 3 days to decrease the anti-nutritional factors, air-sundried for 48 h, and then crushed in a forage machine.

### Chemical analyses

Chemical analyses of representative samples of the experimental ingredients, CFM, and diets were conducted according to (19) for OM (ID number 942.05), EE (using the Soxhlet procedure, ID number 938.06), and CP (as 6.25 × N; ID number 954.01). Neutral detergent fibers (NDF), acid detergent lignin (ADL), and acid detergent fiber (ADF) were evaluated as described previously (20). Cellulose and hemicelluloses were also calculated. The content was calculated as follows: NFE [g/kg dry matter (DM)] = 100 – (CP + ash + EE + CF). All chemical analyses were carried out in duplicate.

### Experimental procedures

Animals in all groups were fed by group feeding, and the tested diets were offered in equal amounts for all groups twice daily at 8 a.m. and 3 p.m., while fresh water was always available. The offered amounts of CFM and roughage were adjusted every 2 weeks according to changes in the animals' body weight. Diets were formulated to meet the requirements of growing goats according to (21).

### Digestibility trials

During the feeding period, three digestibility trials were conducted on three lambs from each group to assess the feeding values and digestibility of the experimental meals. Each digestibility study had a 15-day preparatory phase and a 7-day collection period. As a natural marker, acid-insoluble ash was used (22). During the collection period, feces samples were taken twice daily from the rectum of each animal at 12-h intervals. At the start, middle, and end of the collection period, feedstuff samples were taken. Chemical analyses of the CFM, corn silage, rice straw, and feces samples were carried out using AOAC procedures (2005). The equation proposed by Schneider was used to calculate the nutrient digestibility as follows:


$$\begin{array}{l} \text{Dry matter }(\text{DM})\text{ digestibility},\%\\ \text{=100}-\left[\text{100}\times \frac{\text{AIA}\%\text{ in feed}}{\text{AIA }\%\text{ feces}}\right],\\ \text{Nutrient digestibility},\%\\ \text{=100}-\left[\text{100}\times \frac{\text{AIA}\%\text{ in feed}}{\text{AIA }\%\text{ feces}}\times \frac{\text{Nutrient}\%\text{ in feces}}{\text{Nutrient }\%\text{ feed}}\right], \end{array}$$

where AIA is acid-insoluble ash. Digestible crude protein (DCP) and total digestible nutrients (TDN) were assessed according to the formula in (23).

### Rumen liquor samples

Rumen liquor samples were collected from goats 3 h after morning feeding using a stomach tube and the draw pulse power of an automatic milking machine. Rumen samples were strained using four layers of cheesecloth. The pH of the rumen was measured using an Orian 680 digital PH meter immediately after the samples were extracted. The amount of ammonia nitrogen (NH_3_-N) in the air was measured using the AOAC saturated magnesium oxide distillation method (2005). Total volatile fatty acids in rumen liquor were measured using the steam distillation method described previously (24).

### Blood samples and biochemical analysis

At 3 h after feeding, blood samples were collected from the jugular vein in centrifuge tubes containing EDTA as an anticoagulant. The plasma was then centrifuged for 15 min at 4,000 rotations/min and stored in a deep freezer until analysis. Calorimetric determinations of blood plasma protein, albumin, globulin (by difference), urea nitrogen, AST, and ALT were performed using commercial diagnostic kits.

### Milk yield and composition

Milk production was recorded weekly using the manual milking technique. The udder was stripped completely, and then the total milk production was calculated by collecting milk during the entire experiment period and correcting for 3.5% fat according to (25) as follows: FCM 3.5% fat as FCM (3.5%) = 0.35 M + 18.57 F, where F is the amount of fat in kilograms, and M is the quantity of milk in kilograms. Milk samples were analyzed for total solids, protein, NPN, casein, ash, and fat. The solid-not-fat portion was also determined using the formula reported previously (26).

The carbohydrate content of the milk was calculated by the difference. The Ca and P contents were determined using atomic absorption, according to the method described previously (27). The milk fatty acid composition was determined according to (28). The pH values of the sample were measured using a digital pH meter.

### Feed conversion

The feed conversion efficiency in terms of DM, TDN, and DCP required for 1 kg 3.5% FCM yield was calculated for every animal.

### Economic efficiency

The cost of feed, the feed cost/kg 4% FCM, and the price of 4% FCM were calculated for every goat according to 2020 prices. Additionally, the economic efficiency expressed as the ratio of the price of the 4% FCM yield and the feed cost was estimated.

### Statistical analysis

Data analyses were performed using SPSS version 23.0 software (IBM, New York, NY, USA). The data were statistically analyzed using the general linear model procedure adopted by (29) for one-way ANOVA. A Duncan test was also performed to evaluate the degree of significance among the means. The significance level for all analyses was set at 0.05.

## Results and discussion

The organic matter (OM) content of mango seeds (MS) was found to be approximately similar to that of yellow corn. However, MS was superior in terms of the ether extract (EE), crude fiber (CF), and ash content in comparison with yellow corn. The ash content in yellow corn (1.30%) was slightly lower than the ash content in MS (1.75%), while the nitrogen free extract (NFE) and crude protein (CP) in MS were lower than those in yellow corn (Table 2). Ash, CF, and CP contents in the present study differed from the results found previously (30), where 4.73% ash, 3.98% CF, and 8.98% CP in boiled mango seeds kernel were reported. However, the ash content was 1.40% and 2.38% for yellow corn and MS, respectively (14). The chemical composition of MS in this study differed from that reported by numerous researchers, possibly because those authors used MSK. The present findings are consistent with those previously described (7, 31–33), who reported that MSK is a rich source of carbohydrates (67–82%) and contains moderate amounts of protein (6–10%) and fat (7–14%). However, the present results were inconsistent with the results found by (34). In line with the present study, it was reported that MSK could be a powerful energy source and would probably be a good replacement for maize (33), which is considered the main energy source in the diet of non-ruminants.

**Table 2:** Chemical analysis of CFM ingredients and feedstuffs, and CFM and the tested diets of experimental groups.

**Item**	**DM %**	**Chemical analysis (%, on DM basis)** ^a^					
		**OM**	**CP**	**EE**	**CF**	**NFE**	**Ash**
**CFM ingredients and feedstuffs**							
Yellow corn grains	91.58	98.70	9.29	1.7	2.1	85.7	1.3
Wheat brane	90.50	93.80	14.20	2.7	11.8	65.10	6.2
Undecorticated cotton-seed meal	90.98	93.30	26.40	5.64	24.26	37.00	6.7
Mango seeds	89.04	97.15	8.93	3.5	21.93	62.79	2.85
Soaking mango seeds	88.33	98.21	7.13	4.81	22.21	64.06	1.79
Fresh Berseem	16.31	88.85	16.62	1.21	26.30	44.12	11.15
Wheat Straw	91.75	87.83	2.96	1.59	25.8	57.48	12.17
**Experimental CFM**							
CFM1 (0% MS)	91.23	89.52	14.78	2.94	11.17	60.63	10.48
CFM2 (20% MS)	91.08	89.49	14.66	3.13	12.37	59.33	10.51
CFM3 (40% MS)	90.95	89.47	14.53	3.30	13.59	58.04	10.53
**Experimental diets** ^b^							
G1 (Control, 0% MS)	32.17	89.07	14.34	2.11	18.68	55.51	10.92
G2 (20% MS)	32.16	89.07	14.26	2.21	19.29	53.08	10.94
G3 (40% MS)	32.14	88.99	14.18	2.28	19.93	52.41	10.95

a OM, organic matter; CF, crude protein; CF, crude fiber; NFE, Nitrogen free extract.

b Mango seeds (MS) replaced by yellow corn grains in the concentrate feed mixture (CFM) at the levels of 0 (G1, control), 20 (G2) and 40 % (G3), respectively.

Similar to previous reports, the highest concentration of CF was for CFM3 (40% MS), while the lowest concentration was for CFM1 (0% MS), which may be due to the high CF in MS compared with corn gain (14). The present study also found that replacing corn with MS led to an increase in EE (2.94 to 3.30 g/100 g DM) and CF (11.17 to 13.59 g/100 g DM) in the tested feed mixtures, whereas the nitrogen-free extract content decreased from 60.63 to 58.04 g/100 g DM. These results show that MS is a good source of carbohydrates. The OM, CP, EE, and ash contents were nearly similar for the different diets, whereas CF tended to increase and NFE decrease with increasing MS levels. In line with other research, MS was shown to have greater concentrations of growth energy and CF, because the kernel is usually a significant source of fat, starch, and protein (7, 14). Similarly, it was reported that mango kernels included a higher percentage of fat, starch, and protein (34, 35).

### Fiber fractions

The fiber fractions of the feedstuffs and experimental diets are shown in Table 3. These results show that CFM3 had the highest all-fiber fractions compared with CFM1, except for hemicellulose and non-structural carbohydrates, because MS is superior in terms of CF, NDF, ADF, ADL, and cellulose contents compared with yellow corn. The present study showed variations between the different tested diets in terms of the CF, NFE, NDF, ADF, ADL, CEL, hemicellulose, and non-fiber carbohydrate contents. The present study results suggest that NDF is positively correlated with lignin but negatively correlated with non-fiber carbohydrate. These results are in agreement with those reported previously (36).

**Table 3:** Fiber fractions % of feedstuffs and experimental rations.

**Item**	**NDF**	**ADF**	**ADL**	**Hemi cellulose**	**Cellulose**	**NSC**
**Feedstuffs**					
CFM1	34.76	12.92	4.47	21.84	8.45	39.04
CFM2	35.85	16.30	5.11	19.25	11.19	37.85
CFM3	37.29	19.38	7.35	17.91	12.03	36.35
Fresh berseem	50.51	39.23	8.26	11.34	30.97	20.45
Wheat straw	56.98	38.56	4.15	18.42	34.41	26.30
**Experimental diets** ^a^					
G1 (Control, 0% MS)	43.31	26.01	5.96	17.30	20.06	30.33
G2 (20% MS)	43.86	27.7	6.28	16.10	21.42	29.74
G3 (40% MS)	44.58	29.24	7.39	15.34	21.85	28.97

a Mango seeds (MS) replaced by yellow corn grains in the concentrate feed mixture the (CFM) at levels of 0 (G1, control), 20 (G2) and 40 % (G3), respectively.

NDF, Neutral detergent fiber; ADF, Acid detergent fiber; ADL, Acid detergent lignin.

### Digestibility coefficients and feeding values

The digestibility coefficients of all nutrients (DM, OM, CP, CF, EE, and NFE) and TDN increased (*P* < 0.05) in the diets that included CFMs containing MS in G2 and G3, with the highest observed in G3. The DCP content was nearly similar for the different groups and not significantly affected by feeding of MS (Table 4). This result may imply that a 40% concentration of MS supplementation would support rumen microbial activity fermentation, which, in turn, facilitates improved digestibility. Similarly, it was revealed (37) that a specific minimum daily dry matter intake is required to fulfill an animal's hunger and to allow the digestive tract to function properly. This indicates a healthy source of roughage that could improve rumination and prevent rumen digestive disorders (38). Similarly, it was found (15) that the digestibility coefficients of DM, CP, EE, and CF increased (*P* < 0.05) as the level of MS increased. The digestibility of OM and NFE, and feeding values, as well as the TDN and DCP were higher (*P* < 0.05) in groups fed MS, at 20% in G2 and 40% in G3, compared with G1. Similarly, it was reported (13) that the nutrient digestibility of growing West African dwarf sheep fed a diet containing 75% mango kernel meal was the best in terms of CF, EE, and NFE and differed significantly (*P* < 0.05) from those containing 0, 50, and 100% mango kernel meal. The addition of 25% or 50% yellow corn instead of MSK considerably increased the nutritional digestibility coefficients of DM, OM, CP, CF, and NFE (*P* < 0.05) (14). When MSK was substituted with 25% or 50% yellow corn content in the control ration, the TDN and DCP values improved (*P* < 0.005).

**Table 4:** Digestibility coefficients and feeding values of the tested diets by male kids in the experimental groups.

**Item**	**Experimental diets** ^1^			**SEM**
	**G1** **(0%MS)**	**G2** **(20% MS)**	**G3** **(40% MS)**	
**Digestibility coefficients (%)**				
Dry matter	68.83^b^	71.39^a^	72.82^a^	0.63
Organic matter	70.03^c^	72.03^b^	73.51^a^	0.55
Crude protein	71.17^b^	73.08^a^	73.97^a^	0.49
Ether extract	67.41^c^	70.83^b^	73.23^a^	0.88
Crude fiber	65.69^b^	69.32^a^	70.51^a^	0.75
Nitrogen free extract	71.26^b^	72.78^ab^	74.57^a^	0.61
**Feeding value (%)**				
Total digestible nutrients	63.47^b^	65.45^a^	66.92^a^	0.55
Digestible crude protein	10.28	10.44	10.52	0.05

1 Mango seeds (MS) replaced by yellow corn grains in the concentrate feed mixture (CFM) at levels of 0 (G1, control), 20 (G2) and 40 % (G3), respectively.

abc Means in the same row with different superscripts significantly differ (P < 0.05). SEM, Standard error of the mean.

### Feed intake and feed conversion

The average daily feed intake of goats fed different experimental rations is presented in Table 5. Because the goats were group fed, comparisons regarding the feed intakes of all nutrients are made on a relative basis rather than on a statistical basis. The CFM, fresh berseem, wheat straw, DM, TDN, and DCP intakes tended to increase for goats fed CFMs containing MS and tended to increase as the MS concentration increased from 20 to 40%. These results agreed with previous findings (15), where the average daily feed intake from feedstuffs (CFM, fresh berseem, and wheat straw) or DM, TDN, and DCP was slightly higher in treatment groups containing MS than in the control group. Similarly, significant differences (*P* < 0.05) were observed in the digestible nutrient intake in growing West African dwarf sheep fed different levels of mango kernel meal-based diets (13).

**Table 5:** Average daily feed intake ratio of the tested diets by female kids in the experimental diets.

**Item**	**Experimental diets** ^**1**^			**SEM**
	**G1** **(0% MS)**	**G2** **(20% MS)**	**G3** **(40% MS)**	
**As fed basis (kg/day)**				
CFM (kg)	0.702	0.722	0.737	
Fresh berseem (kg)	3.140	3.227	3.286	
Wheat straw (kg)	0.140	0.143	0.146	
Total	3.982	4.092	4.169	
**On DM basis (kg/day)** ^2^				
DM (kg)	1.281	1.316	1.340	0.027
TDN (kg)	0.813^c^	0.861^b^	0.897^a^	0.012
DCP (kg)	0.132	0.137	0.141	0.002

^1^Mango seeds (MS) replaced by yellow corn grains in the concentrate feed mixture (CFM) at levels of 0 (G1, control), 20 (G2) and 40 % (G3), respectively.

^2^TDN, total digestible nutrients; DCP, Digestible crude protein.

^abc^Means in the same row with different superscripts significantly differ (P < 0.05). SEM, Standard error of the mean.

The results of feed conversion reveal that introducing MS in the CFMs of dairy goats improved feed conversion, and the amounts of DM, TDN, and DCP required per kilogram of 3.5% FCM were lower (*P* < 0.05) in G2 and G3 when compared with G1 (Table 6). These results may be attributed to the improved FCM yield upon feeding with MS. Similar results were previously obtained (15), where the feed conversion in growing lambs improved in lambs fed CFMs containing MS.

**Table 6:** Feed conversion and economic feed efficiency of experimental diets fed by female kids in different groups.

**Item**	**Experimental diets** ^**1**^			**SEM**
	**G1** **(0% MS)**	**G2** **(20% MS)**	**G3** **(40% MS)**	
**Feed conversion (kg/kg 3.5% FCM)**				
Dry matter	1.253^a^	1.135^b^	1.113^b^	0.23
Total digestible nutrients	0.795^a^	0.743^b^	0.745^b^	0.16
Digestible crude protein	0.129^a^	0.118^b^	0.117^b^	0.045
**Economic efficiency**				
Feed cost (L.E./day)	4.85	4.89	4.88	0.18
Feed cost (L.E./kg 3.5% FCM)	4.75^a^	4.22^b^	4.05^b^	0.21
Price of 3.5% FCM (L.E./day)	6.13^b^	6.95^a^	7.22^a^	0.26
Net revenue (LE)	1.28^b^	2.06^a^	2.34^a^	0.12
Relative net revenue (%)	100.00^b^	160.94^a^	182.81^a^	2.32
Economic efficiency	1.26^b^	1.42^a^	1.48^a^	0.03
Relative economic efficiency %	100.00	112.70	117.46	1.17

^1^Mango seeds (MS) replaced by yellow corn grains in the concentrate feed mixture (CFM) at levels of 0 (G1, control), 20 (G2) and 40 % (G3), respectively.

^ab^Means in the same row with different superscripts significantly differ (P < 0.05). SEM, Standard error of the mean.

Prices of diets (L.E./Kg): CFM1: 4.65 L.E., CFM2: 4.50 L.E., CFM3: 4.36 L.E., FB:0.48 L.E., WS: 0.60 L.E.,3.5% FCM: 6 L.E.

### Economic efficiency

The feeding cost was the same for the different groups, whereas the feed cost per 1 kg 3.5% FCM decreased (*P* < 0.05) for G2 and G3 when compared with G1 (Table 6). However, the prices of the 3.5% FCM yield, net revenue, relative net revenue, economic efficiency, and relative economic efficiency were higher (*P* < 0.05) for G2 and G3 compared with G1. The best economic efficiency occurred with the 40% MS replacement of corn compared with all treatments. This result was obtained because MS is a mango, and it is handled at a cheaper rate than yellow corn, in addition to the decreased feed consumption, which may be due to the bitter taste of MS. The relative economic efficiency of 20 and 40% MS replacement of corn increased by approximately 12.70 and 17.46%, respectively, compared with that of the control group. Similarly, it was reported (39) that the feed cost per kilogram weight gain of broilers was lowest in the group fed 10% rice polish as a replacement for corn. Additionally, it was reported (15) that the daily feed cost of growing lambs was nearly similar in all lambs, but the feed cost per kilogram weight gain decreased (*P* < 0.05) with increasing the level of MS. However, the price of daily gain, economic feed efficiency (EFE), and relative EFE increased (*P* < 0.05) with increasing MS level. In line with the present results, the economic analysis revealed a decrease in the total feeding costs and a superior benefit-to-cost ratio (16).

### Rumen liquor parameters

The ruminal pH value and the total volatile fatty acids (TVFA) and NH_3_-N concentrations were not affected by incorporating MS into the diets (Table 7). Similarly, MSK replaced 25 or 50% of the yellow maize in the control ratio which significantly increased the ruminal pH (*P* < 0.05); however, it had no significant (*P* > 0.05) effect on the NH_3_-N and total volatile fatty acid (TVFA) concentrations (14). The high fermentation of mango waste likely stimulated microbial growth, resulting in high NH_3_-N capture by microorganisms (40). The greater proportion of minor VFA is consistent with the higher NH_3_-N concentrations, as minor VFA and NH_3_-N are the final products of protein degradation in the rumen (41).

**Table 7:** Blood parameters of Damascus female goat fed different experimental rations.

**Item**	**Experimental diets** ^**1**^			**SEM**
	**G1** **(0% MS)**	**G2** **(20% MS)**	**G3** **(40% MS)**	
**Rumen liquor parameters**				
pH value	6.19	6.25	6.16	0.05
TVFA's (M_m_/100 ml)	12.41	12.00	12.67	0.35
NH_3_-N(mg/100 ml)	7.84	7.84	7.65	0.57
**Blood plasma biochemical**				
Total protein (g/100 ml)	7.08	6.92	7.15	0.11
Albumin (g/100 ml)	3.42	3.30	3.37	0.08
Globulin (g/100 ml)	3.66	3.62	3.78	0.12
Albumin: Globulin ration	0.93	0.91	0.89	0.04
Cholesterol (mg/dl)	77.00^a^	65.17^b^	63.72^b^	1.89
Creatinine (mg/100 ml)	0.58	0.61	0.59	0.03
AST (IU/l)	75.17^a^	67.33^b^	68.83^b^	2.23
ALT (IU/l)	11.83	11.67	12.33	0.29
Urea-N (mg/100 ml)	56.50	57.83	55.67	4.19

^1^Mango seeds (MS) replaced by yellow corn grains in the concentrate feed mixture (CFM) at levels of 0 (G1, control), 20 (G2) and 40 % (G3), respectively.

^ab^Means in the same row with different superscripts significantly differ (P < 0.05). SEM, Standard error of the mean.

### Blood parameters

Only the cholesterol concentration and AST activity were decreased (P < 0.05) when yellow corn grain was replaced with MS in G2 and G3. Other plasma biochemical parameters, including total protein, albumin, globulin, albumin to globulin ratio, creatinine, ALT, and urea–N were not affected by MS (Table 7). This result agrees with that found previously (42), where 0.28% dietary mango saponin supplementation decreased the plasma total cholesterol content in cockerels. This could be explained by the presence of mangiferin in the mango saponin. The hypocholesterolemia effect of MSK may be related to flavonoid components that may prevent lipid peroxidation, which regulates cholesterol synthesis. Serum creatinine kinase (CK) activity for Gimmizah cockerels decreased (*P* < 0.05) in the treated groups. At 10% MSK substitution with corn, the lowest creatinine kinase (CK) concentration was reported. According to a previous study (43), elevated serum CK levels are linked to cell damage and muscle cell disintegration. The enhancement of muscle cells in that study, by replacing 10 and 15% of the maize in the cockerels' diet with MSK, was linked to phenolic chemicals, which may reduce oxidation reactions in the cell.

### Milk yield and composition

The actual milk and 3.5% FCM yields increased (*P* < 0.05) with increasing MS concentration. G3 (40% MS) exhibited the highest actual milk and 3.5% FCM yields (1.880 and 1.204 kg/day, respectively), followed by G2 (1.650 and 1.159 kg/day, respectively), with G1 presenting the lowest values (1.514 and 1.022 kg/day, respectively). Concerning the milk composition, G2 had significant (*P* < 0.05) TS, TP, NPN, casein, ash, fat, SNF, lactose, and Ca contents as well as fat yield G3, while G1 had the lowest (Table 8). The P content and pH values were nearly similar in all groups. Similarly, it was found (1, 44) that MSK contained considerable levels of calcium, phosphorus, sodium, magnesium, iron, zinc, and copper.

**Table 8:** Milk yield and composition for dairy goats fed different experimental rations.

**Item**	**Experimental diets** ^**1**^			**SEM**
	**G1** **(0% MS)**	**G2** **(20% MS)**	**G3** **(40% MS)**	
**Milk yield (kg/day)** ^2^				
Actual milk	1.514^c^	1.650^b^	1.880^a^	0.093
3.5% FCM	1.022^c^	1.159^b^	1.204^a^	0.072
**Milk composition** ^2^				
TS %	10.43^c^	11.35^a^	10.92^b^	0.02
TP %	2.91^b^	2.99^a^	2.96^ab^	0.025
NPN %	0.35^b^	0.38^a^	0.36^b^	0.009
Casein %	2.01^b^	2.06^a^	2.03^ab^	0.012
Ash %	0.73^b^	0.82^a^	0.79^a^	0.014
Fat %	2.65^c^	3.13^a^	2.94^b^	0.06
SNF %	7.77^c^	8.19^a^	7.96^b^	0.02
Lactose %	4.14^c^	4.41^a^	4.25^b^	0.01
Ca (mg/100g)	123.67^b^	133.00^a^	131.33^a^	1.12
P (mg/100g)	116.00	117.67	116.33	1.27
Fat yield (g/day)	25.76^c^	27.77^a^	26.73^b^	0.18
pH	6.53	6.54	6.54	0.007

^1^Mango seeds (MS) replaced by yellow corn grains in the concentrate feed mixture (CFM) at levels of 0 (G1, control), 20 (G2) and 40 % (G3), respectively.

^2^FCM, fate corrected milk; TS, total solids; NPN, non-protein nitrogen; SNF, solids not fat.

^abc^Means in the same row with different superscripts significantly differ (P < 0.05). SEM, Standard error of the mean.

### Milk fatty acid profile

Feeding the goats with MS increased the concentrations of caprioc, caprylic, capric, stearic, oleic, elaidic, and linoleic acids (Table 9). In contrast, feeding with MS decreased the concentrations of butyric, laueic, tridecanoic, myristic, myristoleic, pentadecanoic, heptadecanoic, cis-10-Heptadecanoic, cis-11-Eicosenoic, linolenic, arachidonic, and lignoseric acids in the milk fat (Table 9). The apparent change in the milk's stearic acid content when the goats were fed with MS may be due to the high percentage of that acid in MS, as explained previously (4). In line with other reports, the assessment of the milk fatty acid profile of goats revealed no difference among treatments (16). However, the level of myristoleic fatty acid C14:1 cis-9 decreased linearly by 0.15 g/100 g of fatty acids for every 1% increase in whole mango meal levels (*P* < 0.05).

**Table 9:** Milk fatty acid profile in dairy goats fed MS replacing corn.

**Fatty acids concentration, %**	**Experimental diets** ^**1**^			**SEM**
	**G1** **(0% MS)**	**G2** **(20% MS)**	**G3** **(40% MS)**	
Butyric acid (C4:0)	0.29^a^	0.12^c^	0.21^b^	0.07
Caproic acid (C6:0)	0.87^c^	1.07^b^	1.32^a^	0.19
Caprylic acid (C8:0)	1.78^c^	2.40^b^	2.60^a^	0.37
Capric acid (C10:0)	9.44^a^	10.06^b^	10.36^a^	0.41
Laueic acid (C12:0)	5.47^a^	4.52^b^	3.92^c^	0.68
Tridecanoic acid (C13:0)	0.19^a^	0.06^b^	0.05^b^	0.07
Myristic acid (C14:0)	11.52^a^	9.74^c^	10.06^b^	0.83
Myristoleic acid methyl ester (14.1)	0.39^a^	0.29^b^	0.20^c^	0.83
Pentadecanoic acid (C15:0)	1.39^a^	0.96^b^	0.68^b^	0.31
Palmitic acid (C16:0)	29.44^a^	25.74^c^	29.02^b^	1.75
Palmitoleic acid (C16:1n7)	0.52^a^	0.49^b^	0.39^c^	0.06
Heptadecanoic acid (C17:0)	0.57^a^	0.50^b^	0.41^c^	0.07
Cis-10-Heptadecanoic acid (C17:1)	0.84^a^	0.73^b^	0.56^c^	0.12
Stearic acid (C18:0)	10.02^c^	13.69^a^	11.73^b^	1.59
Oleic acid (C18:1n9c)	21.10^b^	23.55^a^	20.72^c^	1.33
Elaidic acid (C18:1n9t)	0.71^c^	0.98^b^	1.24^a^	0.23
Linoleic acid (C18:2n6c)	2.43^c^	2.89^b^	3.25^a^	0.36
Linolelaidic acid (C18:2n6t)	0.24^b^	0.37^a^	0.24^b^	0.07
Arachidic acid (20.0)	0.20^b^	0.22^a^	0.15^c^	0.03
γ- Linolenic acid (C18:3n6)	—^c^	0.07^a^	0.04^b^	0.02
Cis-11- Eicosenoic acid(20.1)	0.52^a^	0.46^b^	0.34^c^	0.08
Linolenic acid (C18:3n3)	0.68^a^	0.60^c^	0.65^b^	0.04
Cis−11,14- Eicosadienoic acid (C 20.2)	—^b^	0.06^a^	0.06^a^	0.01
Arachidonic acid (C20: 4n6)	0.23^a^	0.19^b^	0.19^b^	0.02
Eicosapentaenoic acid (C20:5n3)	—^a^	0.04^b^	—^a^	0.02
Lignoseric acid (C24:0)	1.18^a^	0.12^c^	0.98^b^	0.49
Docosahexaenoic acid (C22:6n3)	—^a^	0.05^b^	—^a^	0.02

^1^Mango seeds (MS) replaced by yellow corn grains in the concentrate feed mixture (CFM) at levels of 0 (G1, control), 20 (G2) and 40 % (G3), respectively.

^abc^Means in the same row with different superscripts significantly differ (P < 0.05). SEM, Standard error of the mean.

## Conclusions

The results revealed that replacement of 20–40% of yellow corn grain with mango seeds resulted in no adverse effects on the performance of Damascus goats. In addition, the inclusion of mango seeds at a concentration of 40% improved the digestibility, milk yield, feed conversion, economic efficiency, and milk composition.

## Data availability statement

The original contributions presented in the study are included in the article/supplementary material, further inquiries can be directed to the corresponding authors.

## Ethics statement

This study was conducted according to the research ethics approved by the committee on research of the Animal Production Research Institute, Agricultural Research Center. Written informed consent was obtained from the owners for the participation of their animals in this study.

## Author contributions

HE-S, GE-E, WR, MZ, and MA designed the experiment, carried out the research, and laboratory analysis. AM, AS, ME, LE-N, and PD did the data analysis, wrote the manuscript, and revised the manuscript. All authors reviewed and agreed on the final manuscript.
